# A non-singular fractional-order logistic growth model with multi-scaling effects to analyze and forecast population growth in Bangladesh

**DOI:** 10.1038/s41598-023-45773-1

**Published:** 2023-11-17

**Authors:** Mohammad Sharif Ullah, K. M. Ariful Kabir, Md. Abdul Hakim Khan

**Affiliations:** 1 Department of Mathematics, Feni University, Feni, Bangladesh; 2 Department of Mathematics, Bangladesh University of Engineering and Technology, Dhaka, Bangladesh

**Keywords:** Ecology, Mathematics and computing

## Abstract

This paper is primarily concerned with data analysis employing the nonlinear least squares curve fitting method and the mathematical prediction of future population growth in Bangladesh. Available actual and adjusted census data (1974–2022) of the Bangladesh population were applied in the well-known autonomous logistic population growth model and found that all data sets of the logistic (exact), Atangana-Baleanu-Caputo (ABC) fractional-order derivative approach, and logistic multi-scaling approximation fit with good agreement. Again, the existence and uniqueness of the solution for fractional-order and Hyers-Ulam stability have been studied. Generally, the growth rate and maximum environmental support of the population of any country slowly fluctuate with time. Including an approximate closed-form solution in this analysis confers several advantages in assessing population models for single species. Prior studies predominantly employed constant growth rates and carrying capacity, neglecting the investigation of fractional-order methods. Thus, the current study fills a crucial gap in the literature by introducing a more formal approach to analyzing population dynamics. Therefore, we bank on the findings of this article to contribute to accurate population forecasting and planning, national development, and national progress.

## Introduction

Bangladesh is a small nation in South Asia, ranked 8th largest and the 10th most densely populated country. As the growth rate and size of the population are significant factors for its economy and policy, the resulting parameter of increasing population growth is alarming to Bangladesh’s people and policymakers. Thus, population control should be the requirement’s highest priority in national policy. Predicting and estimating the growth of the country’s future population is one of the most significant facts that can be solved by a mathematical model such as population dynamics. Food supply, available land, technology, birth and death rates, emigration, and prevailing conditions in the country, like war, are critical factors associated with the growth of the population, which also fluctuates with change.

Differential equations formulating the population dynamics mathematical model have an extensive history. To illustrate the individuals as continuous variables who alter the growth of the population in separate time steps that have disclosed exquisite results in many applications, a single-species mathematical population model bears the first-order nonlinear ordinary differential equation with the initial condition ignoring the spatial effects like diffusion and scattering^1–3^. Among other mathematical population models, the nonlinear Verhulst’s logistics model is widely used in population census data for distinct countries^4–7^. However, in 1976, Obaidullah^8^ presented the “Expo-linear model” for Bangladesh’s population growth and explored that this model is a better indicator of projecting population growth over time using either an exponential or linear model. The main limitation of his work is the sensitivity of its basic parameters. If any country’s population growth reaches zero levels, then the total population size does not increase, which helps the policymaker redecorate their long-term future planning. In 1980, Mallik^9^ projected that Bangladesh would attain a zero population growth rate in 2080. If the population growth is more significant than zero, then every year adds a certain number of people to the total population, and additional food is needed. Kabir and Chowdhury^10^ have established a correlation between population augmentation and food exposition in Bangladesh regarding the matter mentioned earlier. In this situation, decision-makers and planners face some additional problems. Karim et al.^11^ also present a comparative analysis of Bangladeshi and Indian population growth and adaptation. Beekman^12^, Haque et al.^13^, Ali et al.^14^, Hossain et al.^15^, Szabo et al.^16^, Mondol et al.^17^, Ullah et al.^18^, and Biswas and Paul^19^ studied the population-changing dynamics of the Bangladesh population and forecast using well-known mathematical and statistical tools. Karim et al.^20^ conducted a comparative analysis of three mathematical models and assessed the accuracy and closeness of these models in predicting the population increase of Bangladesh and India by the decision of the twenty-first century. Thus, overcoming such difficulties in future population predictions for Bangladesh is an inevitable challenge for decision-makers and planners.

Fractional-order modeling has become an indispensable instrument in numerous scientific disciplines due to its ability to characterize complex and nonlocal behaviors that traditional integer-order models cannot adequately capture. Significantly, fractional modeling is required to comprehend and represent systems with memory effects, anomalous diffusion, and complex dynamics. Fractional modeling's significance rests in its ability to provide more accurate and realistic representations of complex systems, thereby advancing our understanding and problem-solving abilities across a wide range of disciplines. In recent decades, mathematical models of integer derivatives have progressed considerably due to the lack of information or the precision with which reality is translated into a mathematical formula. Such models cannot always wholly imitate real-world phenomena. As a result, its utilization is vital to humankind’s prophecy, which helps people comprehend what could ensue shortly and take preventative actions to avert worst-case scenarios. Compared to standard integer-order models, fractional-order (FO) models give a more specific and comprehensive insight into the complicated behavior of many diseases. FO systems are deemed more favorable than integer-order systems due to their inherent characteristics and ability to describe memory^21, 22^. In addition, standard integer-order systems cannot investigate the dynamics between two points. Many ideas and conceptions about FO derivatives have been offered in the literature. The classical FO derivative is shown in^22^ as an example. Current literature^23^ discusses a novel FO derivative and related conclusions based on the generalized exponential rule. As mentioned earlier, the FO derivatives^22–24^ were effectively employed to represent real-world processes in various domains, including biology, engineering, and physics^25–33^. The inability to explain the nonlocal dynamics and crossover behavior of various real-world phenomena can be attributed to the classical FO derivative’s non-singular kernel. In 2016, Atangana and Baleanu^34^ introduced a novel fractional-order (FO) derivative that utilizes a generalized Mittag–Leffler function as a nonlocal and non-singular kernel. This derivative aims to address concerns regarding the non-locality of the kernel in the derivative proposed in a previous study^35^ and to examine the nonlocal complex behavior of diverse systems more effectively. The Atangana-Beleanu derivative, introduced in a recent study^34^, has been applied in various fields^36–43^ to simulate real-world problems.

Many authors have used several fractional derivatives to describe the logistic equation (see^44–47^ for examples), whereas Qureshi et al.^47^ used actual statistical data to investigate the logistic growth model. Kumar et al.^48^ and^49^ extensively examined the logistic model inside a fractional framework, namely, the Caputo-Fabrizio (CF) and Caputo operator, and utilized fixed point theory to establish the uniqueness and stability analysis of the corresponding solution. Noupoue et al.^50^ conducted a study on the fractional order logistic equation. They employed various numerical schemes, including the generalized Euler's method, the power series expansion (Grunwald Letnikov) technique, and the CF approach, and established the existence and uniqueness of the nonlinear logistic equation by utilizing Hadamard fractional derivative and integral formulae. Bas and Ozasalan^51^ used the Atangana-Baleanu-Caputo operator^34^, which is a fractional operator characterized by a nonlocal and non-singular kernel, to investigate some real-world problems, namely, Newton’s law of cooling, population growth, logistic equation, blood alcohol model.

Nevertheless, there are several latent attributes of the ruling model that remain unexplored. It is important to conduct a thorough exploration in order to identify additional characteristics of the model that can contribute to the analysis and study of real-world issues, namely, the population of Bangladesh. In light of this motivation, we use census data and implement the ABC fractional-order method to examine the novel characteristics of the governing model.

Due to the representation of nonlinearity in the governing equations of the population model, the attainment of exact solutions is only feasible to a limited extent. Therefore, one can proceed with the approximate solutions^52^. The present study will further explore perturbation techniques and multi-scale analysis, which involve the introduction of two distinct time scales, namely the fast and ordinary scales, for independent variables. This approach will be utilized to derive an approximate solution for the single-species population model. Details can be seen in^53–57^. In 1999, Meyer^58^ analyzed the attributes pertaining to the dynamic carrying capacity of the logistic model. Furthermore, a novel framework was employed to re-examine two empirical instances of human population expansion. For the first time in 2003 and 2007, Stojkov^59^, Shepherd, and Stojkov^60^ implemented the multi-scaling method in the logistic population model while carrying capacity varying with time, whereas the growth rate is constant. Then, in 2008, Grozdanovski et al.^61^ applied the multi-scaling analysis method to the logistic population model while the growth rate and carrying capacity varied slowly. Stojkov, Shepherd and Stojkov and Grozdanovski et al.^59–61^ did it for an arbitrarily chosen data set, not for a particular region or country. Moreover, in 2015, Dose et al.^62^ applied the multi-scale analysis method featuring a capricious carrying capacity in a discrete population model.

In the case of Bangladesh populations, these previous studies on population models of single species employed constant growth rates and carrying capacity. No fractional-order or multi-scaling scheme is employed for analyzing and predicting the population of Bangladesh. Nevertheless, the changing demographic trend^63–67^ of the population of Bangladesh lets us know that the population size and the growth rate are oscillating over time. Here, we assume both are functions of time for investigating and predicting the population of Bangladesh. As a result, the multi-scaling method is compelling for analyzing and forecasting the population of Bangladesh. Thus, the principal object of this research is to investigate the logistic growth model through the logistic ABC fractional-order derivative approach and the logistic multi-scaling approximation for analyzing and predicting the future population of a particular country, Bangladesh, for 2023 and onwards (2080), using predated actual and adjusted census data from 1974 to 2022.

The development of this work is as follows: the model characteristics are elaborately discussed in “Mathematical model” section. In “Fractional-order analysis of the logistic growth model” section, we present the fractional-order logistic model using ABC fractional derivatives, existence and uniqueness, Hyers-Ulam stability, and numerical technique, where the fractional order of differentiation is $\alpha$. A multi-scaling analysis of the logistic model is described in “Multi-scaling analysis” section. The calibration of the Bangladesh population census data and the determination of carrying capacity, intrinsic growth rate, and other parameters are given in “Logistic model used to fit the census data” section. In “Results and discussion” section, we offer some numerical results through the graphs. The concluding words are given in “Conclusion” section.

## Mathematical model

This research starts with the well-known nonlinear Verhulst logistic population growth model^68^ and the adjustment of the Malthusian model^69^. Additionally, he posited that a population’s growth is contingent upon its size and the carrying capacity of its environment, which refers to the maximum population that the said environment can sustain. Generally, single-species population models often comprise growth rate parameters and carrying capacity. It expresses the evolutionary character of the population. Thus, the model is

1 $$\frac{dN(t)}{dt}=rN\left(t\right)\left(1-\frac{N\left(t\right)}{K}\right), N\left(0\right)={N}_{0}.$$

where $r, K, N(t)$ is the growth rate, maximum environmental support, or carrying capacity, and the total size of the population.

The exact solution of Eq. (1) is

2 $$N\left(t\right)=\frac{K}{1+\left(\frac{K}{N}-1\right){e}^{-rt}}.$$

## Fractional-order analysis of the logistic growth model

Over the last several decades, many researchers and academics have considered fractional calculus, which is based on a fundamental concept that involves considering derivatives and integrals of non-integer orders. As a result, these derivatives and integrals possess additional degrees of freedom. Furthermore, many academics have shown interest in fractional calculus due to the swift advancements and progressions in nano-technology. Therefore, in the case of population dynamics^70–72^, it is crystal clear that the integer form of the logistic model is sufficient for analyzing and predicting any country’s population and able to describe the future population movement fluctuating the value of growth rate, despite the fact that fractional calculus when compared to classical calculus, provides a more comprehensive framework for the exploration of hereditary and memory-related characteristics, as well as distinctive behaviors exhibited by various processes and phenomena. In the same context, using lower-order fractional derivatives, one can easily demonstrate the future population movement of any country’s population. Our predominant aspect is to describe the future population movement of Bangladesh through different order fractional-order derivatives, which assists the policymakers of Bangladesh in taking an impeccable control strategy to control the population growth of the country.

### **Definition 3.1**

^21^ The Riemann–Liouville fractional differential operator for a function $F\left(t\right)$ with order $\alpha >0$ is defined by

3 $${{}_{0}{}^{RL}D}_{t}^{\alpha }F\left(t\right)=\frac{1}{\Gamma \left(n-\alpha \right)}\frac{{d}^{n}}{d{t}^{n}}{\int }_{0}^{t}{\left(t-s\right)}^{n-\alpha -1}F\left(s\right)ds,t>0$$

where $$n-1\le \alpha <n,n\in {\varvec{N}}.$$

### **Definition 3.2**

The well-known Caputo fractional-order derivative^35^ of a function $F\left(t\right)$ with order $\alpha >0$ is as follows,

4 $${{}_{0}{}^{C}D}_{t}^{\alpha }F\left(t\right)=\frac{1}{\Gamma \left(n-\alpha \right)}{\int }_{0}^{t}\frac{{F}^{\left(\alpha \right)}\left(\eta \right)}{(t-\eta {)}^{\alpha -n-1}}d\eta ,t>0,\alpha >0,n-1<\alpha \le n,n\in {\varvec{N}}$$

where, $\Gamma$ is the well-known Gamma function, $\alpha$ is the order of the Caputo fractional derivative operator ${}^{C}D^{\alpha } .$

### **Definition 3.3**

^23^ Consider $F\left(t\right)\in {\mathcal{H}}^{1}\left({c}_{1},{c}_{2}\right),{c}_{1}>{c}_{2}, \alpha \in ]\mathrm{0,1}[,$ then the CF fractional-order derivative of a function $F(t)$ is defined as follows:

5 $${{}_{0}{}^{CF}D}_{t}^{\alpha }F(t)=\frac{\mathfrak{R}\left(\alpha \right)}{1-\alpha }{\int }_{{c}_{1}}^{t}\frac{d}{dv}F(t)exp\left[-\alpha \frac{t-x}{1-\alpha }\right]dv.$$

where $\mathfrak{R}\left(\alpha \right)$ is a normalization function satisfying $\mathfrak{R}\left(0\right)=\mathfrak{R}\left(1\right)=1.$

### **Definition 3.4**

Let us assume the function $F:\left[0,\infty \right]\to {\mathbb{R}}.$ The ‘‘conformable fractional derivative’’ of the function $F\left(t\right),$ $\forall t>0$ denoted by ${T}_{\alpha }\left(F\right)\left(t\right)$ of fractional-order $\alpha \in (\mathrm{0,1})$ is defined as follows:

6 $${T}_{\alpha }\left(F\right)\left(t\right)=\underset{\mathcal{E}\to 0}{\mathrm{lim}}\frac{F\left(t+\varepsilon {t}^{1-\alpha }\right)-F(t)}{\varepsilon }.$$

### **Definition 3.5**

Under the condition of $F(t)\in {\mathcal{H}}^{1}\left(0,T\right),$ the general definition of ${\mathbb{A}}{\mathbb{B}}{\mathbb{C}}$ fractional-order derivative of a function $F(t)$ is as follows:

7 $${{}_{0}{}^{{\mathbb{A}}{\mathbb{B}}{\mathbb{C}}}D}_{t}^{\alpha }F(t)=\frac{{\mathbb{A}}{\mathbb{B}}{\mathbb{C}}\left(\alpha \right)}{1-\alpha }{\int }_{0}^{t}\frac{d}{dv}F(t){\varepsilon }_{\alpha }\left[\frac{-\alpha }{1-\alpha }{\left(t-v\right)}^{\alpha }\right]dv.$$

In Eq. (7), substituting ${\varepsilon }_{\alpha }\left[\frac{-\alpha }{1-\alpha }{\left(t-v\right)}^{\alpha }\right]$ by ${\varepsilon }_{1}=exp\left[\frac{-\alpha }{1-\alpha }(t-v)\right]$ for the Capto-Fabrizo differential operator. On top of that, it is to be noted that ${{}_{0}{}^{{\mathbb{A}}{\mathbb{B}}{\mathbb{C}}}D}_{t}^{\alpha }\left[constant\right]=0.$

The normalization function is denoted by the symbol ${\mathbb{A}}{\mathbb{B}}{\mathbb{C}}\left(\alpha \right)$, and its definition is as follows: ${\mathbb{A}}{\mathbb{B}}{\mathbb{C}}\left(0\right)={\mathbb{A}}{\mathbb{B}}{\mathbb{C}}\left(1\right)=1.$ Additionally, ${\varepsilon }_{\alpha }$ represents a unique function that is referred to as the Mittag–Leffler function.

### **Definition 3.6**

Let us assume that $F(t)$ is a function of the interval $L[0, T]$, then the integral that corresponds to it in the ${\mathbb{A}}{\mathbb{B}}{\mathbb{C}}$ sense is provided by:

8 $${{}_{0}{}^{{\mathbb{A}}{\mathbb{B}}{\mathbb{C}}}I}_{t}^{\alpha }F(t)=\frac{1-\alpha }{{\mathbb{A}}{\mathbb{B}}{\mathbb{C}}\left(\alpha \right)}F(t)+\frac{\alpha }{{\mathbb{A}}{\mathbb{B}}{\mathbb{C}}(\alpha )\Gamma (\alpha )}{\int }_{0}^{t}{\left(t-v\right)}^{\alpha -1}F(v)dv.$$

### Lemma 1

According to proposition 3, described in^28^, the anticipated solution of the supposed problem for the fractional order $0<\alpha \le 1$ is

$${{}_{0}{}^{{\mathbb{A}}{\mathbb{B}}{\mathbb{C}}}D}_{t}^{\alpha }F\left(t\right)=y(t), t\in \left[0,T\right],$$

$$F\left(0\right)={F}_{0}.$$

Considering that the right side disappears at time $t=0$, then

9 $$F\left(t\right)={F}_{0}+\frac{1-\alpha }{{\mathbb{A}}{\mathbb{B}}{\mathbb{C}}\left(\alpha \right)}y(t)+\frac{\alpha }{\Gamma \left(\alpha \right){\mathbb{A}}{\mathbb{B}}{\mathbb{C}}\left(\alpha \right)}{\int }_{0}^{t}{\left(t-v\right)}^{\alpha -1}y\left(v\right)dv.$$

### Fractional order model

This section explores a fractional-order logistic population growth model utilizing the ABC fractional derivative^34^, which can be summarized as follows: the inspiration for this section is derived from the model previously introduced in Sect. "Mathematical Model".

10 $${{}_{0}{}^{{\mathbb{A}}{\mathbb{B}}{\mathbb{C}}}D}_{t}^{\alpha }N\left(t\right)=rN\left(1-\frac{N}{K}\right),$$

under the initial value $N\left(0\right)\ge 0.$

### Existence and uniqueness of the solution for the ABC model

We derive the solution’s existence and uniqueness concerning the Atangana-Baleanu Caputo derivative for the Eq. (10). Consider a continuous real-valued function with the notation $B(K)$ accompanying the supremum-norm characteristic. This function belongs to a Banach space on $H=[0,b]$ and $Q=B[H]$ with the norm $\Vert N\Vert$,

whereas $$\Vert N\Vert =\underset{t\in k}{\mathrm{sup}}\left|N\right|$$.

The Atangana-Baleanu-Caputo fractional integral operator is employed on both sides of Eq. (10), yielding the resultant outcome.

11 $${N\left(t\right)-N\left(0\right)={}_{0}{}^{{\mathbb{A}}{\mathbb{B}}{\mathbb{C}}}D}_{t}^{\alpha }\left\{rN\left(1-\frac{N}{K}\right)\right\}.$$

As per the definition of the Atangana-Baleanu-Caputo (ABC) fractional derivative, it can be observed that

12 $$\begin{aligned} N\left(t\right)-N\left(0\right) & =\frac{1-\alpha }{{\mathbb{A}}{\mathbb{B}}{\mathbb{C}}\left(\alpha \right)}f\left(\alpha ,t,N\left(t\right)\right) \\ & \quad +\frac{\alpha }{\Gamma \left(\alpha \right){\mathbb{A}}{\mathbb{B}}{\mathbb{C}}\left(\alpha \right)}{\int }_{0}^{t}{\left(t-v\right)}^{\alpha -1}f\left(\alpha ,v,N\left(v\right)\right)dv, \end{aligned}$$

where

13 $$f\left(\alpha ,t,N\left(t\right)\right)=rN\left(1-\frac{N}{K}\right).$$

If $N\left(t\right)$ have an upper limit, $f$ must satisfy the Lipschitz condition. Thus, in the context of couple functions $N\left(t\right)$ and ${N}^{*}(t)$, one can write,

$$\Vert f\left(\alpha ,t,N\left(t\right)\right)-f\left(\alpha ,t,{N}^{*}\left(t\right)\right)\Vert =\Vert r(N-{N}^{*})\left(1-\frac{(N-{N}^{*})}{K}\right)\Vert .$$

Let us assume, $\psi =\Vert r\left(1-\frac{(N-{N}^{*})}{K}\right)\Vert .$

Then we can write,

14 $$\Vert f\left(\alpha ,t,N\left(t\right)\right)-f\left(\alpha ,t,{N}^{*}\left(t\right)\right)\Vert \le \psi \left(N\left(t\right)-{N}^{*}\left(t\right)\right),$$

which demonstrates that the Lipschitz criteria is satisfied. The results of applying the expressions in (11) in a recursive way are as follows:

$${N}_{n}\left(t\right)-N\left(0\right)=\frac{1-\alpha }{{\mathbb{A}}{\mathbb{B}}{\mathbb{C}}\left(\alpha \right)}f\left(\alpha ,t,{N}_{n}\left(t\right)\right)+\frac{\alpha }{\Gamma \left(\alpha \right){\mathbb{A}}{\mathbb{B}}{\mathbb{C}}\left(\alpha \right)}{\int }_{0}^{t}{\left(t-v\right)}^{\alpha -1}N\left(\alpha ,v,{N}_{n}\left(v\right)\right)dv.$$

Now, when ${N}_{0}\left(t\right)=N\left(0\right),$ one can write using the differences between consecutive terms

15 $$\begin{aligned} {N}_{n}\left(t\right)&={N}_{n}\left(t\right)-{N}_{n-1}\left(t\right)=\frac{1-\alpha }{{\mathbb{A}}{\mathbb{B}}{\mathbb{C}}\left(\alpha \right)}\left({f}_{1}\left(\alpha ,t,{N}_{n-1}\left(t\right)\right)-f\left(\alpha ,t,{N}_{n-2}\left(t\right)\right)\right) \\ & \quad +\frac{\alpha }{\Gamma \left(\alpha \right){\mathbb{A}}{\mathbb{B}}{\mathbb{C}}\left(\alpha \right)}{\int }_{0}^{t}{\left(t-v\right)}^{\alpha -1}\left(f\left(\alpha ,v,{N}_{n-1}\left(v\right)\right)-f\left(\alpha ,v,{N}_{n-2}\left(v\right)\right)\right)dv. \end{aligned}$$

Here, it is crucial to note that

$${N}_{n}\left(t\right)=\sum_{i=0}^{n}{I}_{{N}_{i}}\left(t\right).$$

On top of that, in the context of Eq. (14), allowing for

$${I}_{{N}_{n-1}}\left(t\right)={{N}_{n-1}\left(t\right)-N}_{n-2}\left(t\right), \;\; one \;\; can\;\; write$$

16 $$\Vert {I}_{{N}_{n}}\left(t\right)\Vert \le \frac{\alpha }{{\mathbb{A}}{\mathbb{B}}{\mathbb{C}}\left(\alpha \right)}{\psi }_{1}\Vert {I}_{{N}_{,n-1}}\left(t\right)\Vert \frac{\alpha }{\Gamma \left(\alpha \right){\mathbb{A}}{\mathbb{B}}{\mathbb{C}}\left(\alpha \right)}{\psi }_{1}{\int }_{0}^{t}{\left(t-v\right)}^{\alpha -1}\Vert {I}_{{N}_{n-1}}\left(v\right)\Vert dv.$$

#### **Theorem 1**

*The fractional-order model denoted by Eq.* (10) *possesses a distinct solution provided that the condition for* $t\in [0,b]$ *is satisfied.*

17 $$\frac{1-\alpha }{{\mathbb{A}}{\mathbb{B}}{\mathbb{C}}\left(\alpha \right)}\Psi +\frac{\alpha }{\Gamma \left(\alpha \right){\mathbb{A}}{\mathbb{B}}{\mathbb{C}}\left(\alpha \right)}{b}^{\alpha }\Psi <1.$$

#### ***Proof***

It is a presumption that the function $N(t)$ is bounded. As a preliminary matter, Eq. (14) clarifies that $f$ is a valid representation of the Lipschitz condition. Therefore, by using Eq. (17) in conjunction with a recursive hypothesis, we arrive at the following:

18 $$\Vert {I}_{{N}_{n}}\left(t\right)\Vert \le \Vert {N}_{0}\left(t\right)\Vert {\left(\frac{1-\alpha }{{\mathbb{A}}{\mathbb{B}}{\mathbb{C}}\left(\alpha \right)}\psi +\frac{\alpha {b}^{\alpha }}{\Gamma \left(\alpha \right){\mathbb{A}}{\mathbb{B}}{\mathbb{C}}\left(\alpha \right)}\psi \right)}^{n}.$$

Therefore, when $n\to \infty ,$ the above sequence exists and hold $\Vert {I}_{{N}_{n}}\left(t\right)\Vert \to 0.$

Furthermore, according to triangle inequality, for any $k$, one can write Eq. (18) as follows:

19 $$\Vert {I}_{{N}_{n+k}}\left(t\right)-{I}_{{N}_{n}}\left(t\right)\Vert \le \sum_{j=n+1}^{n+k}{\Omega }^{\mathrm{j}}=\frac{{\Omega }^{\mathrm{n}+1}-{\Omega }^{\mathrm{n}+\mathrm{k}+1}}{1-\Omega },$$

$$where, \;\; \Omega =\frac{1-\alpha }{{\mathbb{A}}{\mathbb{B}}{\mathbb{C}}\left(\alpha \right)}\Psi +\frac{\alpha {b}^{\alpha }}{\Gamma \left(\alpha \right){\mathbb{A}}{\mathbb{B}}{\mathbb{C}}\left(\alpha \right)}\Psi <1 \;\; by \;\; hypothesis.$$

#### Hyers-Ulam stability

##### **Definition**

^34^ $\forall \delta >0,\,\exists\, constants\, \xi >0,$ proposed model’s ABC fractional-order integral form (10) is called Hyers-Ulam stable, when

20 $$\left|N\left(t\right)-\frac{1-\alpha }{{\mathbb{A}}{\mathbb{B}}{\mathbb{C}}\left(\alpha \right)}f\left(\alpha ,t,N\left(t\right)\right)+\frac{\alpha }{\Gamma \left(\alpha \right){\mathbb{A}}{\mathbb{B}}{\mathbb{C}}\left(\alpha \right)}{\int }_{0}^{t}{\left(t-v\right)}^{\alpha -1}f\left(\alpha ,v,N\left(v\right)\right)dv\right|\le \delta$$

$$\exists \boldsymbol{ }\dot{N}(t)$$ which satisfying

21 $$\dot{N}(t) =\frac{1-\alpha }{{\mathbb{A}}{\mathbb{B}}{\mathbb{C}}\left(\alpha \right)}f\left(t,N\left(t\right)\right)+\frac{\alpha }{\Gamma \left(\alpha \right){\mathbb{A}}{\mathbb{B}}{\mathbb{C}}\left(\alpha \right)}{\int }_{0}^{t}{\left(t-v\right)}^{\alpha -1}f\left(\alpha ,v,\dot{N}\left(v\right)\right)dv,$$

22 $$\mathrm{implies \; that } \;\;\left|N\left(t\right)-\dot{N}\left(t\right)\right|<\delta \xi .$$

##### **Theorem 2**

*According to the criteria* $H$*, the logistic fractional-order model* (10) *presented in this study exhibits Hyers-Ulam stability (HUS).*

##### ***Proof***

The logistic fractional-order population growth model (10) has a specific solution that conforms to Eq. (11) as per Theorem 1. Henceforth, it is possible to compose,

23 $$\begin{aligned} \left|N\left(t\right)-\dot{N}\left(t\right)\right| & \le \frac{1-\alpha }{{\mathbb{A}}{\mathbb{B}}{\mathbb{C}}\left(\alpha \right)}\Vert f(\alpha ,t,N\left(t\right))-f(\alpha ,t,\dot{N}\left(t\right))\Vert \\ & \quad +\frac{\alpha }{\Gamma \left(\alpha \right){\mathbb{A}}{\mathbb{B}}{\mathbb{C}}\left(\alpha \right)}{\int }_{0}^{t}{\left(t-v\right)}^{\alpha -1}\Vert f\left(\alpha ,v,N\left(v\right)\right)-f(\alpha ,v,\dot{N}\left(v\right))\Vert dv \\ & \le \left[\frac{1-\alpha }{{\mathbb{A}}{\mathbb{B}}{\mathbb{C}}\left(\alpha \right)}+\frac{\alpha }{\Gamma \left(\alpha \right){\mathbb{A}}{\mathbb{B}}{\mathbb{C}}\left(\alpha \right)}\right]I\Vert N\left(t\right)-\dot{N}\left(t\right)\Vert .\end{aligned}$$

Assume,

$$I=\delta ,\frac{1-\alpha }{{\mathbb{A}}{\mathbb{B}}{\mathbb{C}}\left(\alpha \right)}+\frac{\alpha }{\Gamma \left(\alpha \right){\mathbb{A}}{\mathbb{B}}{\mathbb{C}}\left(\alpha \right)}=\eta .$$

Then we have

24 $$\Vert N\left(t\right)-\dot{N}\left(t\right)\Vert \le \delta \eta .$$

According to Eq. (24), the ${\mathbb{A}}{\mathbb{B}}{\mathbb{C}}$ fractional-order integral model (8) is Hyers-Ulam stable. The theorem is proven by the fact that the ABC fractional-order model (10) exhibits Hyers-Ulam stability due to those mentioned above.

#### Calculation of coefficients and constants

Equation (10) can be written as

25 $${{}_{0}{}^{{\mathbb{A}}{\mathbb{B}}{\mathbb{C}}}D}_{t}^{\alpha }N\left(t\right)=f(t,N\left(t\right)),$$

$$N\left(0\right)={N}_{0}.$$

Let us assume there exists a non-decreasing function that does not depend on $N$ such that

$$\left|\varphi (t)\right|\le \epsilon , t\in [0,T]$$

Then, the solution of Eq. (25)

26 $${{}_{0}{}^{{\mathbb{A}}{\mathbb{B}}{\mathbb{C}}}D}_{t}^{\alpha }N\left(t\right)=f\left(t,N\left(t\right)\right)+\varphi \left(t\right), t\in \left[0,T\right]$$

is

$$\begin{aligned} N\left(t\right) & ={N}_{0}+\frac{1-\alpha }{{\mathbb{A}}{\mathbb{B}}{\mathbb{C}}\left(\alpha \right)}f\left(t,N\left(t\right)\right)+\frac{\alpha }{\Gamma \left(\alpha \right){\mathbb{A}}{\mathbb{B}}{\mathbb{C}}\left(\alpha \right)}{\int }_{0}^{t}{\left(t-v\right)}^{\alpha -1}f\left(v,N\left(v\right)\right)dv \\ & \quad +\frac{1-\alpha }{{\mathbb{A}}{\mathbb{B}}{\mathbb{C}}\left(\alpha \right)}\varphi \left(t\right)+\frac{\alpha }{\Gamma \left(\alpha \right){\mathbb{A}}{\mathbb{B}}{\mathbb{C}}\left(\alpha \right)}{\int }_{0}^{t}{\left(t-v\right)}^{\alpha -1}\varphi \left(v\right)dv. \end{aligned}$$

From which we have

27 $$\begin{aligned} & \left|N\left(t\right)-\left({N}_{0}+\frac{1-\alpha }{{\mathbb{A}}{\mathbb{B}}{\mathbb{C}}\left(\alpha \right)}f\left(t,N\left(t\right)\right)+\frac{\alpha }{\Gamma \left(\alpha \right){\mathbb{A}}{\mathbb{B}}{\mathbb{C}}\left(\alpha \right)}{\int }_{0}^{t}{\left(t-v\right)}^{\alpha -1}f\left(v,N\left(v\right)\right)dv\right)\right| \\ & \quad \quad \le \left|\frac{1-\alpha }{{\mathbb{A}}{\mathbb{B}}{\mathbb{C}}\left(\alpha \right)}\varphi \left(t\right)\right|+\frac{\alpha }{\Gamma \left(\alpha \right){\mathbb{A}}{\mathbb{B}}{\mathbb{C}}\left(\alpha \right)}{\int }_{0}^{t}{\left(t-v\right)}^{\alpha -1}\left|\varphi \left(v\right)\right|dv \\ & \quad \quad \le \frac{1-\alpha }{{\mathbb{A}}{\mathbb{B}}{\mathbb{C}}\left(\alpha \right)}\epsilon +\frac{{b}^{\alpha }}{\Gamma \left(\alpha \right){\mathbb{A}}{\mathbb{B}}{\mathbb{C}}\left(\alpha \right)}\epsilon \le \frac{\Gamma (\alpha {)+b}^{\alpha }}{\Gamma \left(\alpha \right){\mathbb{A}}{\mathbb{B}}{\mathbb{C}}\left(\alpha \right)}\epsilon \end{aligned}$$

We will use Eq. (27) in the computation of Hyer-Ulam stability.

##### **Theorem 3**

*Equation* (27) *is Hyer-Ulam stable if* $\frac{1-\alpha }{{\mathbb{A}}{\mathbb{B}}{\mathbb{C}}\left(\alpha \right)}\epsilon +\frac{\alpha {b}^{\alpha }}{\Gamma \left(\alpha \right){\mathbb{A}}{\mathbb{B}}{\mathbb{C}}\left(\alpha \right)}\epsilon <1.$

##### ***Proof***

Let $N$ be any solution of Eq. (27) and $\dot{N}$ be a unique solution. Then

$$\begin{aligned} \left|N\left(t\right)-\dot{N}(t)\right| & =\left|N\left(t\right)-\left({N}_{0}+\frac{1-\alpha }{{\mathbb{A}}{\mathbb{B}}{\mathbb{C}}\left(\alpha \right)}f\left(t,\dot{N}\left(t\right)\right)+\frac{\alpha }{\Gamma \left(\alpha \right){\mathbb{A}}{\mathbb{B}}{\mathbb{C}}\left(\alpha \right)}{\int }_{0}^{t}{\left(t-v\right)}^{\alpha -1}f\left(v,\dot{N}\left(v\right)\right)dv\right)\right| \\ & \le \left|N\left(t\right)-\left({N}_{0}+\frac{1-\alpha }{{\mathbb{A}}{\mathbb{B}}{\mathbb{C}}\left(\alpha \right)}f\left(t,N\left(t\right)\right)+\frac{\alpha }{\Gamma \left(\alpha \right){\mathbb{A}}{\mathbb{B}}{\mathbb{C}}\left(\alpha \right)}{\int }_{0}^{t}{\left(t-v\right)}^{\alpha -1}f\left(v,N\left(v\right)\right)dv\right)\right| \\ & \quad +\left|\frac{1-\alpha }{{\mathbb{A}}{\mathbb{B}}{\mathbb{C}}\left(\alpha \right)}\left(f\left(t,\dot{N}\left(t\right)\right)-f\left(t,N\left(t\right)\right)\right)\right|+\frac{\alpha }{\Gamma \left(\alpha \right){\mathbb{A}}{\mathbb{B}}{\mathbb{C}}\left(\alpha \right)}{\int }_{0}^{t}{\left(t-v\right)}^{\alpha -1}\left|f\left(v,\dot{N}\left(v\right)\right)-f\left(v,N\left(v\right)\right)\right|dv \\ & \le \frac{\Gamma (\alpha {)+b}^{\alpha }}{\Gamma \left(\alpha \right){\mathbb{A}}{\mathbb{B}}{\mathbb{C}}\left(\alpha \right)}\epsilon +\frac{1-\mathrm{\alpha }}{{\mathbb{A}}{\mathbb{B}}{\mathbb{C}}\left(\alpha \right)}\psi \Vert N\left(t\right)-\dot{N}\left(t\right)\Vert +\frac{{b}^{\alpha }}{\Gamma \left(\alpha \right){\mathbb{A}}{\mathbb{B}}{\mathbb{C}}\left(\alpha \right)}\psi \Vert N\left(t\right)-\dot{N}\left(t\right)\Vert \end{aligned}$$

which implies that

$$\Vert N\left(t\right)-\dot{N}\left(t\right)\Vert \le \frac{\frac{\Gamma (\alpha {)+b}^{\alpha }}{\Gamma \left(\alpha \right){\mathbb{A}}{\mathbb{B}}{\mathbb{C}}\left(\alpha \right)}\epsilon }{1-\frac{\Gamma (\alpha {)+b}^{\alpha }}{\Gamma \left(\alpha \right){\mathbb{A}}{\mathbb{B}}{\mathbb{C}}\left(\alpha \right)}\psi }.$$

$$\mathrm{Note\;\; that}, \;\;1-\frac{\Gamma (\alpha {)+b}^{\alpha }}{\Gamma \left(\alpha \right){\mathbb{A}}{\mathbb{B}}{\mathbb{C}}\left(\alpha \right)}\psi \ne 0 \mathrm{i}.\mathrm{e}.,1>\frac{\Gamma (\alpha {)+b}^{\alpha }}{\Gamma \left(\alpha \right){\mathbb{A}}{\mathbb{B}}{\mathbb{C}}\left(\alpha \right)}\psi .$$

Therefore, Eq. (27) is Hyer-Ulam stable.

Now, if $f\left(t,N\right)=rN\left(1-\frac{N}{K}\right),$ one can write

$$\begin{aligned} \left|f\left(t,N\right)-f(t,\dot{N})\right| & =\left|rN\left(1-\frac{N}{K}\right)-r\dot{N}\left(1-\frac{\dot{N}}{K}\right)\right|, \\ & \le r\left|N-\dot{N}\right|+\frac{r}{K}\left|{N}^{2}-{\dot{N}}^{2}\right|, \\ & \le r\left|N-\dot{N}\right|+\frac{2r{N}_{0}}{K}\left|N-\dot{N}\right| if \left|N\right|\le {N}_{0}, \\ & \le \left(r+\frac{2r{N}_{0}}{K}\right)\left|N-\dot{N}\right|, \\ & \le\Psi \left|N-\dot{N}\right|, \end{aligned}$$

where $$\Psi =r+\frac{2r{N}_{0}}{K}.$$

In the case of Bangladesh population-adjusted census data,

$$r=0.0374286, \;\; K=225062093.9465337, \;\; {N}_{0}=76398000$$

$$\therefore\Psi =0.0374286+\frac{2\times 0.0374286\times 76398000}{225062093.9465337}=0.06283730012.$$

Now, if $b=10,\alpha =\frac{1}{2}$ then ${\mathbb{A}}{\mathbb{B}}{\mathbb{C}}\left(\alpha \right)=1.$

Therefore,

$$\frac{\Gamma (\alpha {)+b}^{\alpha }}{\Gamma \left(\alpha \right){\mathbb{A}}{\mathbb{B}}{\mathbb{C}}\left(\alpha \right)}\psi =\frac{\Gamma \left(\frac{1}{2}\right){+10}^\frac{1}{2}}{\Gamma \left(\frac{1}{2}\right)}\times 0.06283730012=0.17494684266<1.$$

Hence, Eq. (27) is Hyer-Ulam stable.

### Numerical analysis

This section will outline the methodology for developing a numerical scheme to solve nonlinear fractional-order logistic differential equations that incorporate fractional derivatives and nonlocal, non-singular kernels. In order to accomplish this task, we shall examine the ordinary nonlinear equation of fractional order, as presented below:

28 $$\left\{\begin{array}{l}{{}_{0}{}^{{\mathbb{A}}{\mathbb{B}}{\mathbb{C}}}D}_{t}^{\alpha }x\left(t\right)=f(t,x\left(t\right)),\\ x\left(0\right)={x}_{0}.\end{array}\right.$$

The fundamental theorem of fractional calculus exemplifies that Eq. (28) can be transformed into a fractional integral equation as follows:

29 $$\begin{aligned} x\left(t\right)-x\left(0\right) & =\frac{1-\alpha }{{\mathbb{A}}{\mathbb{B}}{\mathbb{C}}\left(\alpha \right)}f\left(t,x\left(t\right)\right) \\ & \quad +\frac{\alpha }{\Gamma \left(\alpha \right){\mathbb{A}}{\mathbb{B}}{\mathbb{C}}\left(\alpha \right)}{\int }_{0}^{t}{\left(t-v\right)}^{\alpha -1}f\left(v,x\left(v\right)\right)dv. \end{aligned}$$

One can be written the Eq. (27) at the point ${t}_{n+1},n=\mathrm{0,1},2,\dots$ as follows

30 $$\begin{aligned} x\left({t}_{n+1}\right)-x(0) &=\frac{1-\alpha }{{\mathbb{A}}{\mathbb{B}}{\mathbb{C}}\left(\alpha \right)}f\left({t}_{n},x\left({t}_{n}\right)\right)+\frac{\alpha }{\Gamma \left(\alpha \right){\mathbb{A}}{\mathbb{B}}{\mathbb{C}}\left(\alpha \right)}{\int }_{0}^{{t}_{n+1}}{\left({t}_{n+1}-v\right)}^{\alpha -1}f\left(v,x\left(v\right)\right)dv \\ &=\frac{1-\alpha }{{\mathbb{A}}{\mathbb{B}}{\mathbb{C}}\left(\alpha \right)}f\left({t}_{n},x\left({t}_{n}\right)\right)+\frac{\alpha }{\Gamma \left(\alpha \right){\mathbb{A}}{\mathbb{B}}{\mathbb{C}}\left(\alpha \right)}\sum_{k=0}^{n}{\int }_{{t}_{k}}^{{t}_{k+1}}{\left({t}_{k+1}-v\right)}^{\alpha -1}f\left(v,x\left(v\right)\right)dv. \end{aligned}$$

Using a two-step Lagrange polynomial interpolation, one can estimate the function $f\left(v,x\left(v\right)\right),$ in the interval $\left[{t}_{k},{t}_{k+1}\right]$ as follows:

31 $$\begin{aligned} {P}_{k}\left(v\right) & =\frac{v-{t}_{k-1}}{{t}_{k}-{t}_{k-1}}f\left({t}_{k},x\left({t}_{k}\right)\right)+\frac{v-{t}_{k}}{{t}_{k}-{t}_{k-1}}f\left({t}_{k-1},x\left({t}_{k-1}\right)\right) \\ &=\frac{f\left({t}_{k},x\left({t}_{k}\right)\right)}{h}(v-{t}_{k-1})+\frac{f\left({t}_{k-1},x\left({t}_{k-1}\right)\right)}{h}(v-{t}_{k}) \\ & \simeq \frac{f\left({t}_{k},{x}_{k}\right)}{h}\left(v-{t}_{k-1}\right)+\frac{f\left({t}_{k-1},{x}_{k-1}\right)}{h}\left(v-{t}_{k}\right). \end{aligned}$$

Thus, Eq. (26) can be written as

32 $$\begin{aligned} {x}_{n+1} & ={x}_{0}+\frac{1-\alpha }{{\mathbb{A}}{\mathbb{B}}{\mathbb{C}}\left(\alpha \right)}f\left({t}_{n},x\left({t}_{n}\right)\right) \\ & \quad +\frac{\alpha }{\Gamma \left(\alpha \right){\mathbb{A}}{\mathbb{B}}{\mathbb{C}}\left(\alpha \right)}\sum_{k=0}^{n}\left(\frac{f\left({t}_{k},{x}_{k}\right)}{h}{\int }_{{t}_{k}}^{{t}_{k+1}}\left({v-t}_{k-1}\right){\left({t}_{n+1}-v\right)}^{\alpha -1}dv \right. \\ & \quad \left.-\frac{f\left({t}_{k-1},{x}_{k-1}\right)}{h}{\int }_{{t}_{k}}^{{t}_{k+1}}\left({v-t}_{k}\right){\left({t}_{n+1}-v\right)}^{\alpha -1}dv \right).\end{aligned}$$

Let us assume,

33 $$\begin{aligned} & {\int }_{{t}_{k}}^{{t}_{k+1}}\left({v-t}_{k-1}\right){\left({t}_{n+1}-v\right)}^{\alpha -1}dv ={A}_{\alpha ,k,1}, \\ & {\int }_{{t}_{k}}^{{t}_{k+1}}\left({v-t}_{k}\right){\left({t}_{n+1}-v\right)}^{\alpha -1}dv ={A}_{\alpha ,k,2}. \end{aligned}$$

Then we have

34 $$\begin{aligned} {A}_{\alpha ,k,1} & ={h}^{\alpha +1}\frac{{\left(n+1-k\right)}^{\alpha }\left(n-k+2+\alpha \right)-{\left(n-k\right)}^{\alpha }(n-k+2+2\alpha )}{\alpha (\alpha +1)}, \\ {A}_{\alpha ,k,2} &={h}^{\alpha +1}\frac{{\left(n+1-k\right)}^{\alpha +1}-{\left(n-k\right)}^{\alpha }(n-k+1+\alpha )}{\alpha (\alpha +1)}. \end{aligned}$$

Substituting the value of Eqs. (33) and (34) in Eq. (32), we have

35 $$\begin{aligned}{x}_{n+1}&={x}_{0}+\frac{1-\alpha }{{\mathbb{A}}{\mathbb{B}}{\mathbb{C}}\left(\alpha \right)}f\left({t}_{n},x\left({t}_{n}\right)\right)+\frac{\alpha {h}^{\alpha }}{\Gamma \left(\alpha +2\right){\mathbb{A}}{\mathbb{B}}{\mathbb{C}}\left(\alpha \right)}\sum_{k=0}^{n}\left(\vphantom{\left({\left(n+1-k\right)}^{\alpha +1}-{\left(n-k\right)}^{\alpha }(n-k+1+\alpha )\right)}f\left({t}_{k},{x}_{k}\right)\left({\left(n+1-k\right)}^{\alpha }\left(n-k+2+\alpha \right)\right.\right. \\ & \quad \left.\left.-{\left(n-k\right)}^{\alpha }\left(n-k+2+2\alpha \right)\right)-f\left({t}_{k-1},{x}_{k-1}\right)\left({\left(n+1-k\right)}^{\alpha +1}-{\left(n-k\right)}^{\alpha }(n-k+1+\alpha )\right)\right).\end{aligned}$$

Then, for the Bangladesh population, one can write

36 $$\begin{aligned}{N}_{n+1}& = {N}_{0}+\frac{1-\alpha }{{\mathbb{A}}{\mathbb{B}}{\mathbb{C}}\left(\alpha \right)}f\left({t}_{n},N\left({t}_{n}\right)\right)+\frac{\alpha {h}^{\alpha }}{\Gamma \left(\alpha +2\right){\mathbb{A}}{\mathbb{B}}{\mathbb{C}}\left(\alpha \right)}\sum_{k=0}^{n}\left(\vphantom{\left({\left(n+1-k\right)}^{\alpha +1}-{\left(n-k\right)}^{\alpha }\left(n-k+1+\alpha \right)\right)}f\left({t}_{k},{N}_{k}\right)\left({\left(n+1-k\right)}^{\alpha }\left(n-k+2+\alpha \right)\right.\right. \\ & \quad \left.\left.-{\left(n-k\right)}^{\alpha }\left(n-k+2+2\alpha \right)\right)-f\left({t}_{k-1},{N}_{k-1}\right)\left({\left(n+1-k\right)}^{\alpha +1}-{\left(n-k\right)}^{\alpha }\left(n-k+1+\alpha \right)\right)\right).\end{aligned}$$

## Multi-scaling analysis

To perform a multi-scaling analysis, let us assume all parameters are a function of time. Therefore, an explicit solution of Eq. (1) is written

37 $$N(t) = \frac{{N_{0} e^{{\int\limits_{0}^{t} {r(q)\,dq} }} }}{{1 + N_{0} \int\limits_{0}^{t} {\frac{r(q)}{{K(q)}}e^{{\int\limits_{0}^{q} {(r(s)\,ds} }} dq} }},$$

where $q$ and $s$ are integrating variables.

Firstly, Eq. (37) may only be appraised for a minimal choice of $r(t)$ and $K(t)$ ($r$ and $K$ being positive constants). Secondly, to work out Eq. (1) or Eq. (37), approximate methods (numerical techniques) must be used.

**Figure 1:**
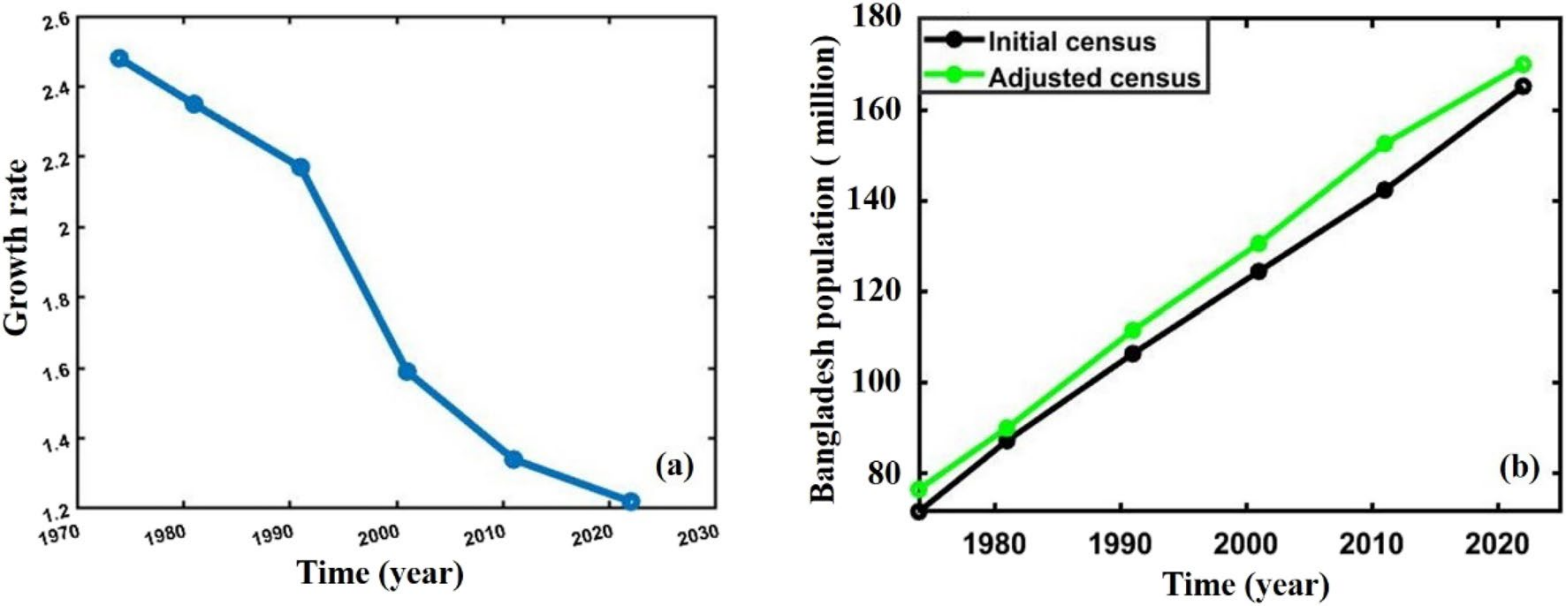
**(a)** Growth rate and **(b)** census data of Bangladesh population.

The actual and adjusted census data^63–67^ (1974–2022) in Fig. 1a,b of the population of Bangladesh, population size, and growth rate $r(t)$ vary with time $t$. Thus, the carrying capacity $K$ slowly changes. The phenomenon arises as a result of gradual oscillations in either the population of a given species, the surrounding environment, or a combination of both factors. Hence, the multi-scaling method becomes convenient for the analysis of the population of Bangladesh.

Therefore, Eq. (1) is no longer autonomous as the variables depend on time $t$; for more details, see^61^. From Eq. (1), one can write,

38 $$\frac{dN\left(t,\varepsilon \right)}{dt}=r\left(\varepsilon t\right)N\left(t,\varepsilon \right)\left(1-\frac{N\left(t,\varepsilon \right)}{K\left(\varepsilon t\right)}\right),N\left(t=0,\varepsilon \right)={N}_{0}.$$

The main goal is to illuminate the procedure, which may always be valid $t\ge 0$, fabricating an approximation to the solution of Eq. (38).

Note: Bangladesh has a long practice of population census. The first census was carried out in 1974 after Bangladesh’s independence (1971). Consequently, the census was made in 1981, 1991, 2001, 2011, and 2022. Therefore, we started our work in 1974, and the corresponding value was calculated using the interpolation method.

### The multi-scale logistic equation

The two-term expansion of the slowly varying logistic model^61^ for analyzing and predicting the population of Bangladesh are as follows:

39 $$N\left(t,\varepsilon \right)=\frac{K\left(\varepsilon t\right){N}_{0}{K}_{0}}{{N}_{0}{K}_{0}-K\left(\varepsilon t\right)\left({N}_{0}-{K}_{0}\right){e}^{-{t}_{0}}}-\frac{\varepsilon {N}_{0}^{2}\left\{\mathop K\limits^{{\prime}} \left(\varepsilon t\right){K}_{0}^{2}{r}_{0}-{K}^{2}\left(\varepsilon t\right) \mathop K\limits^{{\prime}}r\left(\varepsilon t\right){e}^{-{t}_{0}}\right)}{r\left(\varepsilon t\right){r}_{0}{\left\{{N}_{0}{K}_{0}+K\left(\varepsilon t\right)\left({K}_{0}-{N}_{0}\right){e}^{-{t}_{0}}\right\}}^{2}}+\dots$$

Along with $K(\varepsilon t)$ and $r(\varepsilon t),$ which vary periodically, such as

40 $$K\left(\varepsilon t\right)={K}_{0}+\delta sin\varepsilon t \;\;\; \mathrm{and} \;\;\;r\left(\varepsilon t\right)={r}_{0}+\Delta sin\varepsilon t,$$

which affords an approximate solution owing to slowly varying parameters. Here $\delta$ and $\Delta$ are the amplitudes of the oscillatory components when $\varepsilon$ is small. At this juncture, the carrying capacity and growth rate exhibit fluctuations in proximity to their initial values ${K}_{0}$ and ${r}_{0},$ which are emblematic of surroundings that vary slowly with time.

## Logistic model used to fit the census data

In 1838, Verhulst^68^ utilized a logistic model to fit Belgian population census data. Verhulst also described how the vital parameters $r$ and $K$ (for multi-scaling ${r}_{0}$ and ${K}_{0}$) could be estimated from the population data. Therefore,

41 $$K=\frac{{N}_{0}{N}_{1}^{2}+{N}_{1}^{2}{N}_{2}-2{N}_{0}{N}_{1}{N}_{2}}{{N}_{1}^{2}-{N}_{0}{N}_{2}}$$

42 $$r=\frac{1}{T}\mathrm{log}\left[\frac{\frac{1}{{N}_{0}}-\frac{1}{K}}{\frac{1}{{N}_{1}}-\frac{1}{K}}\right]$$

### “Actual” carrying capacity, intrinsic growth rate, and other parameters

This section elaborately discusses calculating the Bangladesh population’s crucial parameters, carrying capacity, and intrinsic growth rate. Usually, population size and growth in a country like Bangladesh straightforwardly impact national plans and natural resources. As a result, the key parameters that control the total population size are the most significant factors in analyzing and predicting the future population of any country. Bangladesh’s census data^63–67^ Fig. 1a,b reveals that the growth of Bangladesh’s population is neither a constant, sinusoidal, nor lines-decaying function. However, it is a strictly decreasing function. As the growth curve strictly decreases, the total population must increase to the carrying capacity value and slow down^68^.

**Figure 2:**
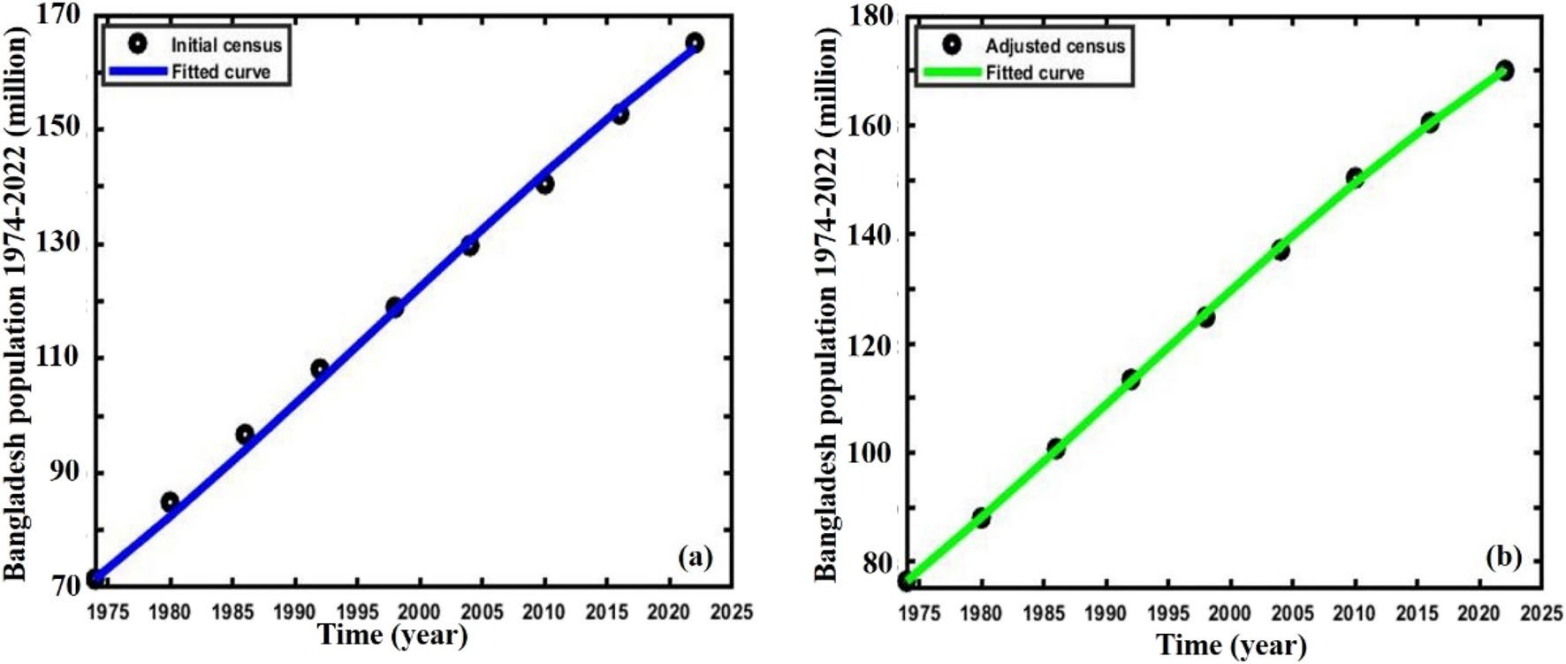
Data fitting curve of **(a)** initial and **(b)** adjusted census data of Bangladesh population.

The Bangladesh population’s^63–67^ initial carrying capacity and intrinsic growth rates are calculated employing actual and adjusted census data using Verhulst’s formula^18, 68^. Then, the nonlinear least square curve fitting method via the “trust-region-reflective algorithm” was used to fit Bangladesh in both the data, Fig. 2a,b, subfigures (a) blue, and (b) green line, which gives the fitted value of two crucial parameters that are illustrated in Table 1. Furthermore, for multi-scaling cases, the values of $\varepsilon , \delta ,\Delta$ are chosen arbitrarily for analyzing the mentioned data and applied for forecasting, Table 2.

**Table 1:** Initial and fitted value of growth rate and carrying capacity of Bangladesh (initial and adjusted) population (1974–2022).

Country	Initial value of $K$	Initial value of $r$	Fitted $K$	Fitted $r$
Bangladesh (initial)	230,343,707.2780429	0.036006736285777	230,343,677.2780429	0.0356036
Bangladesh (adjusted)	230,305,033.7788595	0.036182814238104	225,062,093.9465337	0.0374286

**Table 2:** Value of $\varepsilon , \delta , \Delta$ Bangladesh (initial and adjusted) population.

Cases	$\varepsilon$	$\delta$	$\Delta$
Bangladesh (initial census)	0.1	0.2	0.002120635
Bangladesh (adjusted census)		0.7	0.00160095945

## Results and discussion

**Figure 3:**
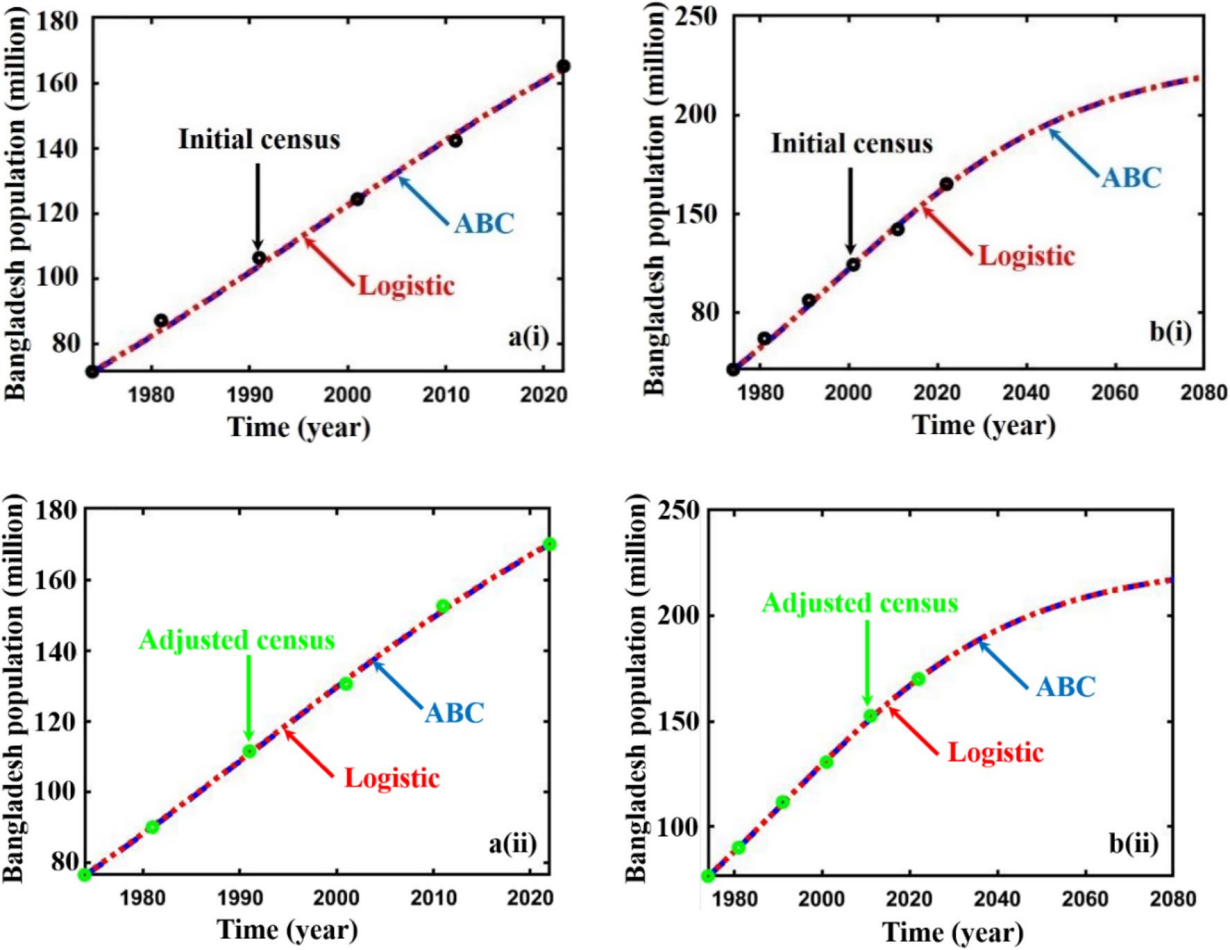
Analysis and projected population of Bangladesh (initial & adjusted). The black and green dot curve represents Bangladesh’s initial and adjusted census data. The red and blue color represents logistic and numerical (ABC) results in all cases. *i and *ii specify the analyzing and predicting results.

**Figure 4:**
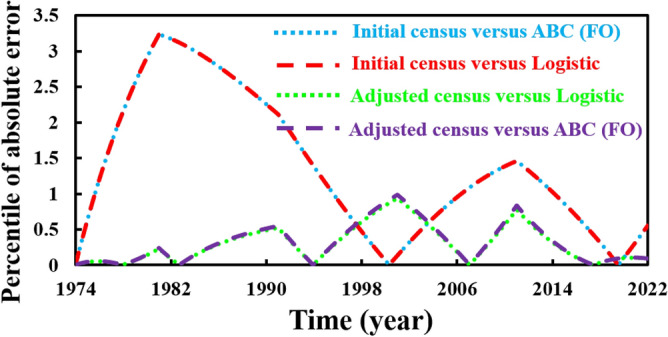
Absolute error curve (census versus logistic and census versus ABC (FO)) of Bangladesh (initial & adjusted) population (1974–2022).

Finally, we forecasted the population of Bangladesh (2023–2080) using the logistic, ABC FO derivative, and logistic multi-scaling approximation method through mathematical simulation, considering that both vital parameters are periodic functions of time for the multi-scaling case. In order to conduct an analysis and make predictions regarding the future population of Bangladesh, it is necessary to ascertain both parameters: the population growth rate and the country’s carrying capacity. Based on the actual and adjusted census data (1974–2022) of the population of Bangladesh^63–67^, according to^18, 68^, the approximate growth rate and carrying capacity are determined. Then, the nonlinear least square curve fitting method via the “trust-region-reflective algorithm” (Fig. 2a,b) has been applied and approximately gives the fitted value $r$ and $K$(for multi-scaling case, it be ${r}_{0}$ and ${K}_{0}$), which are listed in Table 1. The data fitting curve demonstrated in Fig. 2a,b revealed that the analyzed result fits satisfactorily with actual and adjusted census data of the Bangladesh population. One of the exciting features is that Fig. 2b fits more than Fig. 2a. Thus, we can continue analyzing and forecasting the population of Bangladesh. Panels a* and b* of Fig. 3 illustrate the analysis and prediction of the results of the Bangladesh population (initial and adjusted) census data case. $Figures a\left(i\right)$ and $b(i)$ depict the analyzing results of the census (initial and adjusted), logistic solution, and ABC-FO numerical solution, which shows that all data sets are worked with census data with excellent agreement. All line graphs are almost overlapped with each other. All data sets regression coefficients $r$ and ${R}^{2}$ at 95% CI levels are listed in Table 3, which reveals that all the data cases work have an excellent agreement. The absolute error curve of all data cases is demonstrated in Fig. 4. The error curve shows that errors are in a considerable margin, even less, which justifies the current study’s exactness. On top of that, the error is less in the Bangladesh-adjusted census data case compared to the initial census data case. Therefore, the present study works significantly for the Bangladeshi population in both data cases. Therefore, we can continue our efforts to forecast the future population of Bangladesh.

**Table 3:** Regression coefficient and ${R}^{2}$ value of Bangladesh (initial & adjusted) population different data sets in ABC FO case.

Bangladesh population	Regression coefficient, $r$	${R}^{2}$
Initial census vs. exact solution	0.999	0.998
Initial census vs. ABC FO solution	0.999	0.998
Adjuster census vs. Exact solution	0.999	0.999
Adjuster census vs. ABC FO solution	0.999	0.999

**Figure 5:**
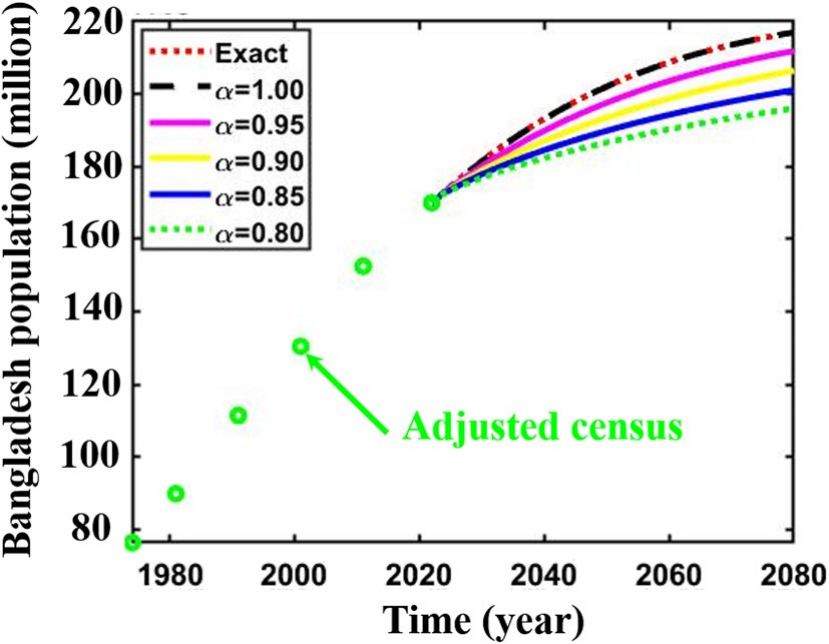
Adjusted census and projected population of Bangladesh (1974–22,080) for different fractional-order $\alpha =\mathrm{1.00,0.95,0.90,0.85,0.80}.$

**Figure 6:**
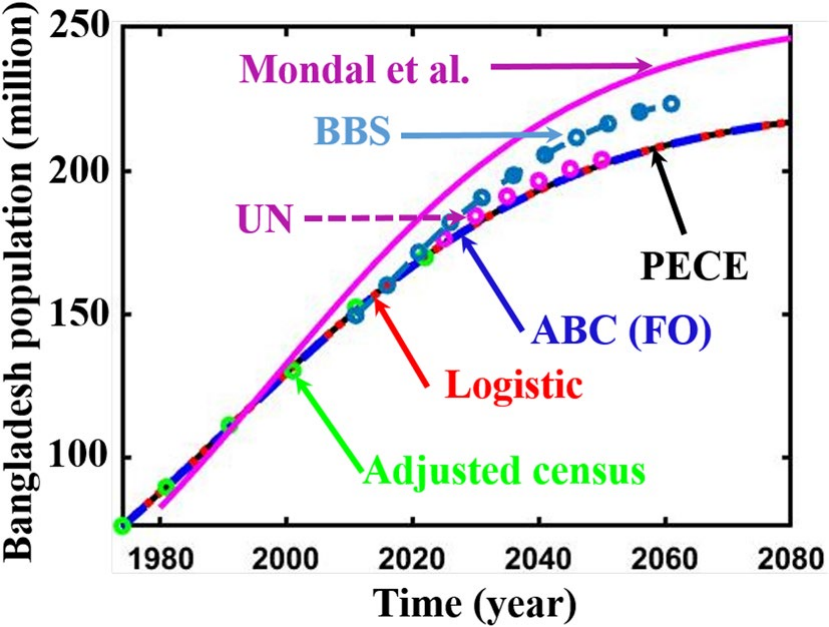
Comparison curve of Bangladesh population (1974–2080) (adjusted census data).

Finally, we forecasted the population of Bangladesh (2023–2080) employing the logistic growth model, where $Figures a\left(ii\right),$ and $b(ii)$ portrayed the predicted results of Bangladesh’s population, which illustrates that gradually Bangladesh’s population size approaches its environmental maximum capacity size, namely the carrying capacity. Finally, Fig. 5 exemplifies the projected population of Bangladesh (2023–2100) for different fraction-order $\alpha =\mathrm{1.0,0.95,0.9,0.85,0.8}.$ If policymakers reduce the growth rate of Bangladesh’s population to a reduced fractional order, then Bangladesh’s future population size will be as shown in Fig. 5. Again, Fig. 6 represents the comparison of the results of the present study with UN^73^, BBS^74^, and Mondal et al.^17^ projected results, which clarify that the present study results follow the adjusted census data case in a better argument compared to other projected results. On top of that, the UN^73^ and current studies’ projected results are in good agreement.

**Figure 7:**
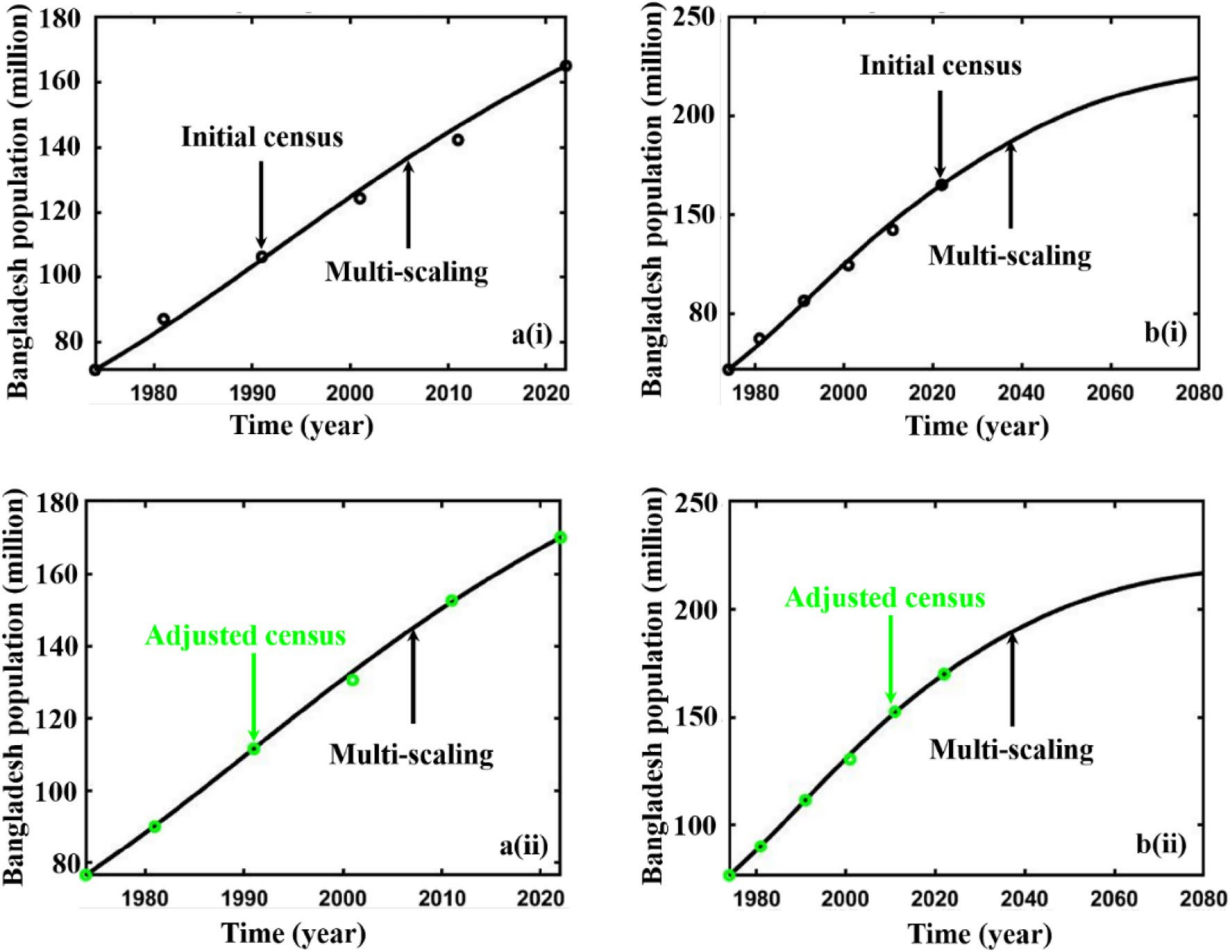
Analysis and projected population of Bangladesh (initial and adjusted). The black and green dot curve represents Bangladesh’s initial and adjusted census data. The black color represents multi-scaling results in both cases. *i and *ii specify both cases analyzing and predicting results.

**Figure 8:**
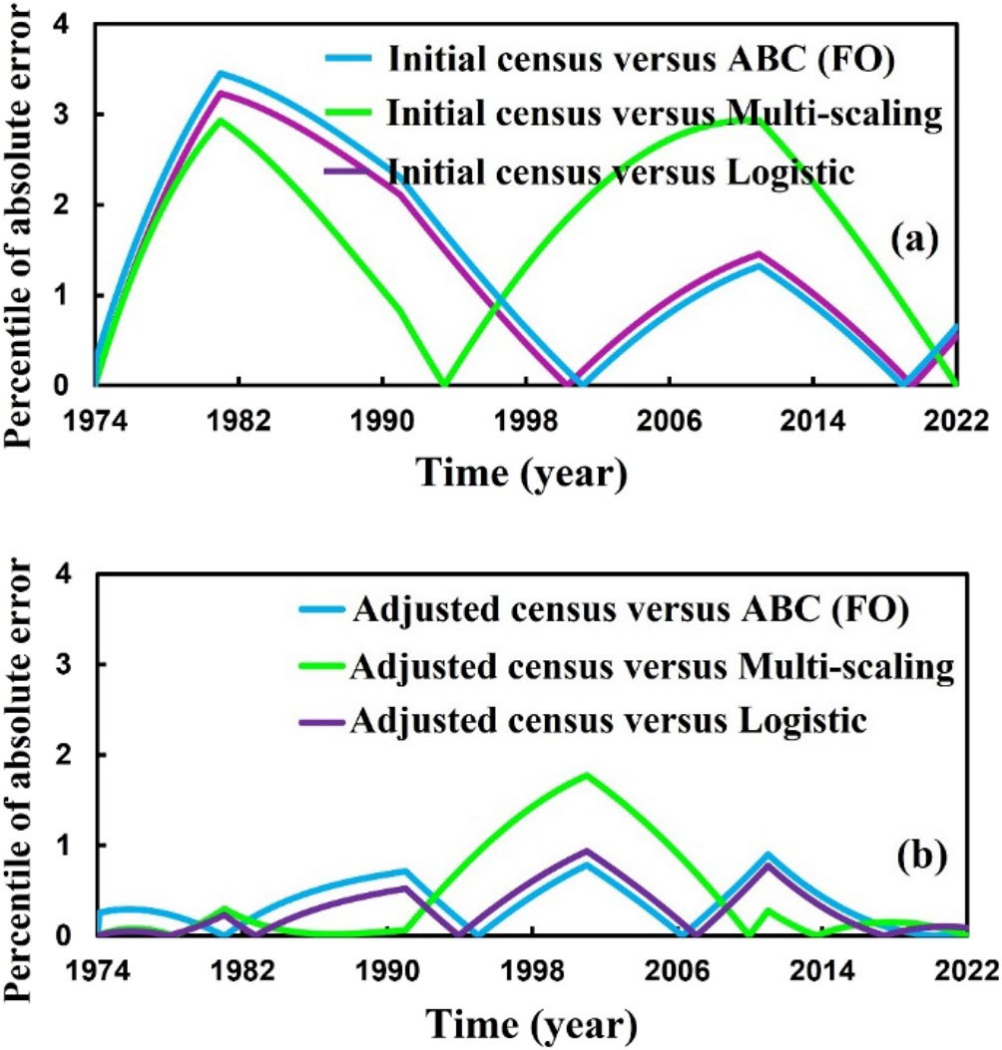
Error curve of Bangladesh (initial and adjusted) population.

**Figure 9:**
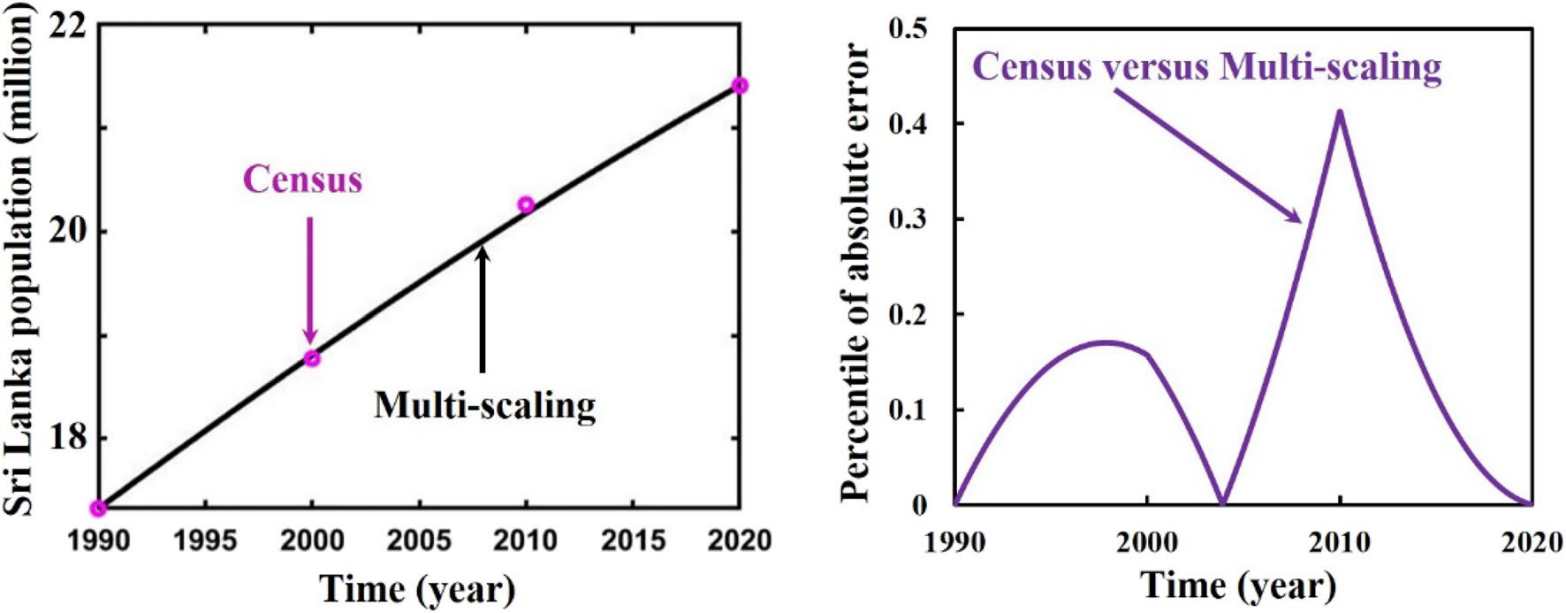
Multi-scaling analysis of Sri Lanka (census) population (1990–2020).

As a final point, we applied a multi-scaling approximation scheme for analyzing and predicting Bangladesh’s (initial and adjusted) population, as demonstrated in Panels a* and b*of Fig. 7. Both data sets regression coefficients, $r$ and ${R}^{2}$ at 95% CI levels are listed in Table 4, which reveals that both data cases are in excellent agreement as logistic and ABC-FO schemes. The absolute error curve of both data cases is demonstrated in Fig. 4. The error curve illustrated in Fig. 8 shows that errors are in a significant margin, even less, which validates the current study’s accuracy. On top of that, the error is less in the Bangladesh-adjusted census data case compared to the initial census data case as previously. In the usual way, one can see that the usual logistic method, the ABC fractional-order logistic method, and the multi-scaling scheme results have no difference; all are identical. Nevertheless, for the analyzing case, the comparison Tables 5, 6 (including logistic model numerical solution multistep Adams–Bashforth-Moulton Predictor–Corrector (PECE) method with the Runge–Kutta-Fehlberg method) illustrated that among other scheme results, the multi-scaling results are more identical and, more precisely, are the same as census data if we consider last year’s data for both data sets. To verify the multi-scaling scheme’s well-posedness, we employ the same strategy for census data of Sri Lanka’s (1990–2020) population^75^ and find that it works favorably. Analyzing result and error curve is depicted in Fig. 9, and different schemes comparing results are listed in Table 7. Therefore, it is easy to say that multi-scaling schemes are more reliable than other methods for analyzing and predicting any country’s population. Therefore, we are banking on this research work to provide a more reliable prediction for the future population measurement of Bangladesh.

**Table 4:** Regression coefficient and ${R}^{2}$ value of Bangladesh (initial & adjusted) different population data sets multi-scaling case.

Bangladesh population	Regression coefficient, $r$	${R}^{2}$
Initial census vs. multi scaling	0.999	0.999
Adjuster census vs. multi scaling	0.999	0.999

**Table 5:** Comparison of different schemes results of Bangladesh population initial census in the year 2022.

Bangladesh initial census	165,158,616.0000	
Scheme	Population size	Percentile of error
Exact (logistic)	164,250,406.1566	0.55%
Predictor–corrector	164,250,406.1568	0.55%
Fractional-order	164,082,545.7138	0.65%
Multi-scaling	165,158,616.0541	$0.3*{10}^{-7}\%$

**Table 6:** Comparison of different schemes results of Bangladesh population adjusted census in the year 2022.

Bangladesh adjusted census	170,000,000.0000	
Scheme	Population size	Percentile of error
Logistic	170,145,565.0601	0.09%
Predictor–corrector	170,145,565.0603	0.09%
Fractional-order	169,990,102.5184	0.006%
Multi-scaling	170,000,000.0009	$0.5*{10}^{-9}\%$

**Table 7:** Comparison of different schemes results of Sri Lanka population census in the year 2020.

Sri Lanka census	21,413,249	
Scheme	Population size	Percentile of error
Logistic	21,438,307.51789	0.12%
Predictor–corrector	21,438,307.51789	0.12%
Fractional-order	21,426,601.72682	0.06%
Multi-scaling	21,413,249.35605	$0.2*{10}^{-5}\%$

**Figure 10:**
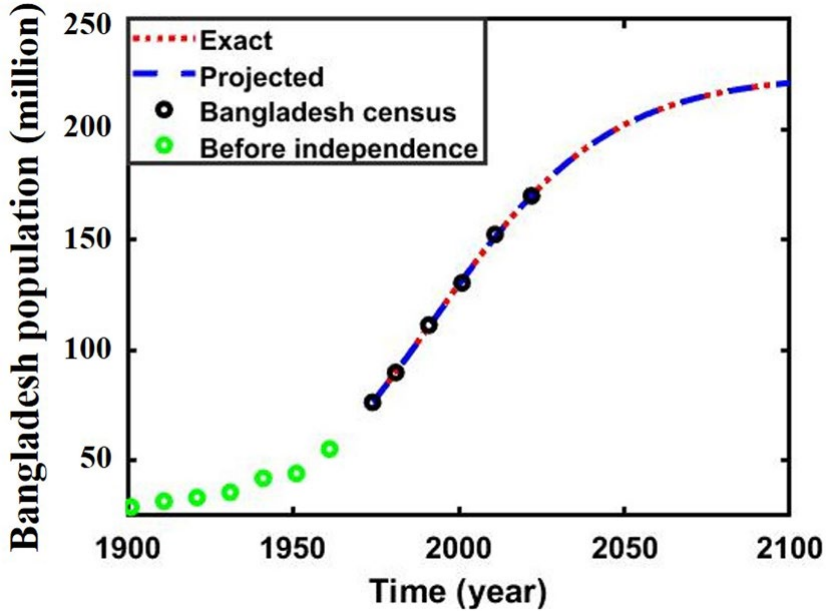
Adjusted census and predicted population of Bangladesh (1901–2080).

On the other hand, the curves, adjusted census, and predicted (1901–2100) demonstrated in Fig. 10 bear a striking resemblance to a sigmoid curve, an S-shaped, well-known main characteristic of the logistic growth model. Again, the fractional-order method is a better choice for its heredity and memory qualities, whereas the multi-scaling logistic approximation models are helpful for mathematicians when no exact data is available to compare with numerical data. Due to the increasing demographic trend, Bangladesh will reach the pinnacle of population size and achieve a zero growth rate. Then, gradually, Bangladesh’s population will decrease like a sigmoid curve, an S-shaped curve^68^.

**Figure 11:**
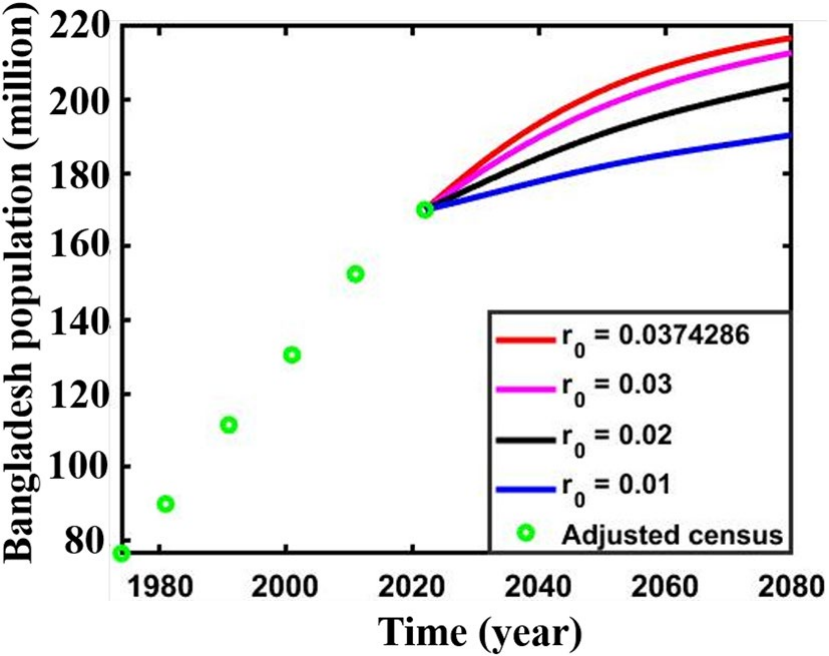
Adjusted census (1974–2022) and predicted (2023–2080) Bangladesh population for different values of ${r}_{0}$.

In conclusion, it is evident that according to^17, 73, 74^ and the present research work predicting results, the People’s Republic of Bangladesh will shortly face some environmental catastrophes, according to Verhulst^68^. It is an alarming threat to our forthcoming generation. Thus, the Government of Bangladesh must take preventive measures and plan to avoid such senile outcomes, which is only possible if the Government of Bangladesh can control the value of growth parameters. On top of that, future demographic projections will offer an estimation of the future populace, which may be enhanced through the mitigation of population growth and the implementation of potential measures. For this purpose, we illustrate Fig. 11 for different values of the growth parameter ${r}_{0}$(the initial growth value of multi-scaling case) where other parameters are the same as previous. The projected final population size (FPS) of adjusted census data results in 2080 is demonstrated in Table 8.

**Table 8:** Predicted population of Bangladesh (2080) for different values of ${r}_{0}$ (adjusted census multi-scaling case).

Adjusted census case (multi-scaling)	
$r\left(\varepsilon t\right)={r}_{0}+\Delta sin\varepsilon t$	Final population size (FPS) (million)
${r}_{0}=0.0374286$	216.887
${r}_{0}=0.03$	212.776
${r}_{0}=0.02$	204.131
${r}_{0}=0.01$	190.396

## Conclusion

Population dynamics models are characterized by significant non-Markovian properties, displaying behavior that is influenced by memory effects. This research study aims to examine the logistic growth model in the context of population dynamics, using both classical (multi-scaling) and non-classical (fractional) differential operators. The predominant objective of this investigation is to analyze and predict the expansion of the population of Bangladesh by utilizing the ABC fractional-order derivative and a multi-scaling logistic approximation model approach while taking into account census data spanning from 1974 to 2022. The fractional-order method is better for its heredity and memory qualities, and one can easily demonstrate the delaying effects through lower-order fractional value, whereas in the conventional process, one has to fluctuate the value of the growth rate to do this. In contrast, multi-scaling logistic approximation models are helpful for mathematicians when no exact data is available to compare with numerical data. Here, the ABC FO derivative and the multi-scale technique have successfully been applied to the logistic growth model in the Bangladesh population (1974–2022), in which the defined parameters of the multi-scaling scheme are changed slowly with time. Our model fitted well with both census data cases (Bangladesh-actual and adjusted census). Therefore, one can conclude that the ABC FO derivative and the multi-scaling logistic aspect worked well for analysis and population forecasting. Although we are investigating only the populations of Sri Lanka and Bangladesh, the theoretical framework can extend to the population growth of arbitrary countries. Thus, we expect this study will bring attention to policymakers and support the government in controlling population growth.

## Data Availability

The datasets used and/or analyzed during the current study are available from the corresponding author upon reasonable request.
